# hnRNP U在急性髓系白血病中临床意义及致病机制研究

**DOI:** 10.3760/cma.j.issn.0253-2727.2022.09.006

**Published:** 2022-09

**Authors:** 春丽 徐, 玙 陈, 婷婷 朱, 振江 孙, 剑虹 储

**Affiliations:** 1 苏州大学医学部造血干细胞移植研究所、苏州大学附属第一医院、江苏省血液研究所、血液学协同创新中心，苏州 215000 Institute of Blood and Marrow Transplantation, Medical College of Soochow University, The First Affiliated Hospital of Soochow University, Jiangsu Institute of Hematology, Collaborative Innovation Center of Hematology, Suzhou 215000, China; 2 皖南医学院第二附属医院血液科，芜湖 241000 Department of Hematology, The Second Affiliated Hospital of Wannan Medical College, Wuhu 241000, China

**Keywords:** hnRNP U, 白血病，髓系，急性, DDR通路, hnRNP U, Leukemia, myeloid, acute, DDR pathway

## Abstract

**目的:**

研究RNA结合蛋白核不均一核糖核蛋白U（hnRNP U）在急性髓系白血病（AML）中临床意义及致病机制。

**方法:**

基于数据库（GEPIA）和本中心数据比较hnRNP U在AML患者及健康对照者中表达情况；通过Cbioportal数据库下载Beat AML数据集（158例），按照hnRNP U表达水平分为高表达组（89例）和低表达组（69例）并对两组临床特征进行比较；选择hnRNP U高表达的Kasumi-1和MOLM-13细胞系，敲低hnRNP U后通过CCK-8检测细胞增殖能力，利用Annexin Ⅴ-APC/7-AAD抗体检测细胞凋亡情况，通过定量分析DNA含量（PI染色）检测细胞周期变化及克隆形成实验检测细胞集落形成能力等方面研究hnRNP U对人AML细胞系生物学行为的影响；利用Western blot法研究敲低hnRNP U后对DDR（DNA Damage Response）通路蛋白cleaved-PARP、p-H2A.X表达的影响。

**结果:**

①泛癌分析发现hnRNP U在AML中高表达，AML患者外周血单个核细胞中hnRNP U mRNA表达水平显著高于健康对照者（0.0315±0.0042对0.0195±0.0006，*P*<0.01）；②hnRNP U高表达组中位发病年龄为56（2～87）岁，hnRNP U低表达组中位发病年龄为65（8～85）岁，hnRNP U高表达组较低表达组发病年龄更早（*t*＝−2.681，*P*＝0.007），且合并FLT3突变比例更高（*χ*^2^＝4.069，*P*＝0.044）；③敲低hnRNP U后Kasumi-1、MOLM-13细胞增殖受抑，细胞凋亡率增高，集落形成能力减弱，细胞周期阻滞在G_2_/M期，与对照组比较，差异均有统计学意义（*P*值均<0.05）；④敲低hnRNP U可引起DDR通路上cleaved-PARP、p-H2A.X蛋白表达上调。

**结论:**

hnRNP U在AML中高表达，敲低hnRNP U后可抑制AML的发生发展，其可能是通过激活DDR通路发挥作用。

## Abstract (en)

**Objective:**

To investigate the clinical significance and pathogenesis of heterogeneous nuclear ribonucleoprotein U（hnRNP U）in acute myeloid leukemia（AML）.

**Methods:**

The expression of hnRNP U, an RNA binding protein, in patients with AML and healthy controls was compared based on the Gene Expression Profiling Interactive Analysis database and the data of the center. The Beat AML Dataset（*n*＝158）was downloaded from the cBioPortal database. The hnRNP U expression level was divided into the high-expression group（*n*＝89）and low-expression group（*n*＝69）, and patients' clinical characteristics were compared. The effect of hnRNP U on the biological behavior of human AML cell lines was studied by Cell Counting Kit-8 assay to detect cell proliferation. Annexin Ⅴ-APC/7-AAD antibodies were used to detect cell apoptosis. DNA content（PI staining）was quantitatively analyzed to detect cell cycle changes, and colony formation experiments were performed to detect cell cloning formation ability after hnRNP U knockdown in Kasumi-1 and MOLM-13 cells. To study the effect of hnRNP U knockdown on the DNA damage response（DDR）pathway proteins of cleaved-PARP, immunoblot analysis using p-H2A.X was conducted.

**Results:**

①Pan-cancer analysis showed that hnRNP U was highly expressed in patients with AML, and the expression level of hnRNP U mRNA in peripheral blood mononuclear cells was significantly higher in patients with AML than in healthy controls（0.0315±0.0042 *vs* 0.0195±0.0006, respectively, *P*<0.01）. ②The age of onset was 56（2–87）years in the high-expression group and 65（8–85）years in the low-expression group（*t*＝−2.681, *P*＝0.007）. Moreover, the high-expression group had a higher proportion of combined FLT3 mutations than the low-expression group（*χ*^2^＝4.069, *P*＝0.044）. ③Compared with the negative control, hnRNP U knockdown inhibited the proliferation（*P*<0.001 and *P*<0.001）, promoted the apoptosis（*P*<0.01 and *P*<0.001）, decreased the colony formation ability（*P*<0.001 and *P*<0.001）, and arrested the cell cycles in the G_2_/M phase（*P*<0.05 and *P*<0.01）of Kasumi-1 and MOLM-13 cells, respectively. ④hnRNP U knockdown could increase the protein expression of cleaved-PARP and p-H2A.X on the DDR pathway.

**Conclusion:**

hnRNP U is highly expressed in AML, and hnRNP U knockdown can inhibit the occurrence and development of AML possibly through the activation of the DDR pathway.

急性髓系白血病（AML）是一种由造血干祖细胞恶性增殖而来的血液系统肿瘤，中位发病年龄在60～70岁[1]。随着新型药物制剂的开发应用及异基因造血干细胞移植技术的发展，AML的诊断和治疗都取得了重大进展，但其预后仍然很差，5年平均生存率为30％[2]。AML分子机制复杂，不同亚型之间异质性大[3]–[4]，因此，寻找AML靶点并研究其作用机制，对于开发新的靶向药物具有重要的指导价值。本课题组前期研究发现，核不均一核糖核蛋白U（heterogeneous nuclear ribonucleoprotein U, hnRNP U）在AML中高表达，本研究拟探讨hnRNP U在AML中的临床意义及致病机制，为AML分子生物学发病机制的研究及靶向治疗提供理论基础。

## 材料与方法

1. 试剂与仪器：ABI 7300型实时定量PCR仪购自美国ABI公司，BCA蛋白定量试剂盒购自美国Thermo Fisher公司，Tanon 5200全自动化学发光成像分析系统和ECL化学发光试剂盒均购自中国上海天能科技有限公司，流式细胞仪（ACEA NovoCyte）购自艾森生物（杭州）有限公司，Allegra X-15R台式冷冻离心机购自美国贝克曼库尔特有限公司，RIPA蛋白裂解液购自碧云天生物技术有限公司，细胞凋亡试剂盒、细胞周期检测试剂盒均购自杭州联科技术股份有限公司，TRIzol总RNA抽提试剂、HiScript^®^ Ⅲ 1st Strand cDNA Synthesis Kit和NovoStart^®^ SYBR qPCR SuperMix Plus试剂盒均购自诺唯赞生物科技有限公司，CCK-8试剂盒购自美国APExBIO公司，RPMI 1640培养基购自美国Gibco公司。兔抗人hnRNP U（16365-1-AP）抗体购自美国Proteintech公司，兔抗人PARP（9542S）抗体以及兔抗人p-H2A.X（9718S）抗体购自美国CST公司，鼠抗人β-actin（ab008-040）以及辣根过氧化物酶（HRP）标记山羊抗兔、鼠抗体均购自杭州联科技术股份有限公司，实验相关引物由苏州金唯智生物科技有限公司合成。

2. 细胞培养: 人AML细胞系U937、OCI-AML3、THP-1、MV4-11、MOLM-13、Kasumi-1由苏州大学附属第一医院陈苏宁教授惠赠，培养于含有10％胎牛血清、100 µg/ml青霉素和100 µg/ml链霉素的RPMI 1640培养基中，37 °C、5％ CO_2_、95％湿度培养箱中培养，平均每2 d换液1次，由本实验室对细胞进行传代培养。

3. 使用慢病毒构建敲低hnRNP U的细胞株：选择的敲低载体为pLKO.1-TRC-GFP，酶切位点选择XbaⅠ和Age Ⅰ，shhnRNP U#1、shhnRNP U#2的敲低引物序列如表1所示，将shRNA引物退火处理，将退火产物与经Xba Ⅰ和Age Ⅰ酶切后的载体进行连接反应，构建相应干扰质粒，选择状态良好的293T细胞包装慢病毒，48 h后收集病毒上清感染Kasumi-1和MOLM-13细胞株，感染72 h后使用流式细胞仪检测慢病毒的感染效率，使用荧光定量PCR技术和Western blot法分别在mRNA和蛋白水平上鉴定敲低hnRNP U的效率。

**表1:** hnRNP U的shRNA干扰序列

引物名称	引物序列（5′→3′）
shhnRNP U#1正向引物	CCGGCAGTGCTTCTTCCCTTACAATCTCGAGATTGTAAGGGAAGAAGCACTGTTTTTG
shhnRNP U#1反向引物	AATTCAAAAACAGTGCTTCTTCCCTTACAATCTCGAGATTGTAAGGGAAGAAGCACTG
shhnRNP U#2正向引物	CCGGGCAACTGTGAGACTGAAGATTCTCGAGAATCTTCAGTCTCACAGTTGCTTTTTG
shhnRNP U#2反向引物	AATTCAAAAAGCAACTGTGAGACTGAAGATTCTCGAGAATCTTCAGTCTCACAGTTGC

4. 实时荧光定量PCR检测hnRNP U mRNA表达水平：使用TRIzol法提取细胞总RNA，并用HiScript^®^ Ⅲ 1st Strand cDNA Synthesis Kit将RNA反转录成cDNA，使用NovoStart^®^ SYBR qPCR SuperMix Plus试剂盒进行相对定量检测，以β-actin为内参，以2^−∆∆Ct^法计算出hnRNP U基因的相对表达量，定量PCR引物序列如表2所示。

**表2:** 实时荧光定量PCR检测hnRNP U表达引物序列

基因	正向引物序列（5′→3′）	反向引物序列（5′→ 3′）
hnRNP U	AAGTATAGCAGAGCCAAATCTCC	CATTGTAAGGGAAGAAGCACTG
β-actin	CCATCATGAAGTGTGACGTGG	GTCCGCCTAGAAGCATTTGCG

5. Western blot法检测hnRNP U、PARP、p-H2A.X蛋白表达水平：使用RIPA蛋白裂解液制备细胞总蛋白并使用BCA蛋白定量试剂盒测定蛋白浓度。每孔使用等量蛋白（30 µg）进行SDS-PAGE，然后将分离的蛋白条带转移至0.45 µm的PVDF膜上，5％脱脂牛奶封闭后，一抗4 °C孵育过夜，二抗室温孵育1 h。使用ECL化学发光试剂盒处理PVDF膜，Tanon全自动化学发光成像分析系统成像。

6. CCK-8法检测细胞增殖能力：以转染后第5天为时间起点，将Kasumi-1和MOLM-13细胞密度调整为5×10^3^/ml，接种于96孔板，每孔100 µl，利用CCK-8试剂检测第1、3、5、7天4个时间点的细胞增殖能力，对应时间段每孔加入10 µl CCK-8试剂，以每个时间点在450 nm处的吸光度（*A*_450_）表示细胞的增殖能力。

7. Annexin Ⅴ-APC/7-AAD双染色法检测细胞凋亡水平：收集对照组（shscramble）、shhnRNP U#1、shhnRNP U#2组的Kasumi-1和MOLM-13细胞，1 300 r/min离心5 min（离心机半径为207.8 mm），弃上清，按细胞凋亡试剂盒说明书操作加入Annexin Ⅴ-APC/7-AAD抗体双标记，避光染色后上流式细胞仪检测细胞凋亡。以早期和晚期凋亡细胞占总细胞数的百分比计算细胞凋亡率。

8. PI染色法检测细胞周期：收集shscramble、shhnRNP U#1、shhnRNP U#2组的Kasumi-1和MOLM-13细胞，1300 r/min离心5 min（离心机半径为207.8 mm），弃上清。上流式细胞仪检测细胞周期，用FlowJo分析软件进行细胞DNA含量分析。

9. 集落形成实验：将0.66％的琼脂糖液和2×RPMI 1640（加入2×抗生素和20％的胎牛血清）等比例混合，轻柔混匀后，取3 ml加入6孔板中。细胞离心计数，每孔接种3 000个细胞，培养箱中培养2周后，结晶紫染色，计数集落形成数量。

10. 统计学处理：使用SPSS 25.0、GraphPad Prism 9.0分析软件进行统计学分析和制图。实验重复3次，计量资料使用均数±标准差表示，计量资料符合正态分布的采用*t*检验，不符合正态分布的采用秩和检验，率的比较采用卡方检验，相关性采用Pearson相关系数表示，*P*<0.05表示差异具有统计学意义。

## 结果

一、hnRNP U在AML患者中表达情况分析

1. hnRNP U表达水平泛癌分析：利用GEPIA在线数据库（http://gepia2.cancer-pku.cn）分析收录的33种肿瘤组织与癌旁正常组织的hnRNP U mRNA表达水平，如图1所示，结果显示hnRNP U在AML中表达高于其他肿瘤以及正常组织。

**图1:**
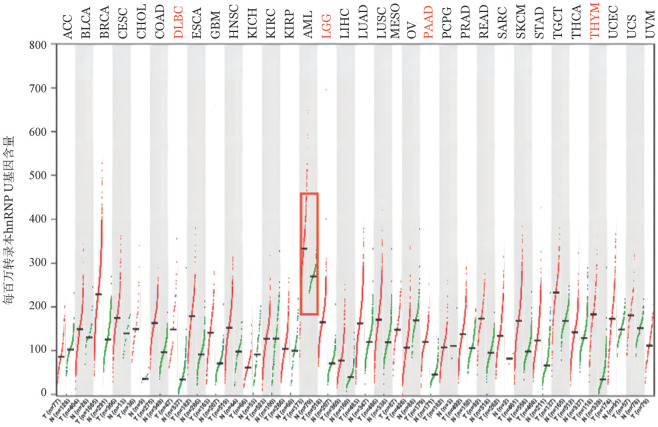
GEPIA数据库中hnRNP U基因在不同肿瘤类型中的表达 绿色代表hnRNP U在正常组织中的表达，红色代表hnRNP U在肿瘤组织中的表达。ACC：肾上腺皮质癌；BLCA：膀胱尿路上皮癌；BRCA：乳腺浸润癌；CESC：宫颈鳞癌；CHOL：胆管癌；COAD：结肠癌；DLBC：弥漫性大B细胞淋巴瘤；ESCA：食管癌；GBM：多形成性胶质细胞瘤；HNSC：头颈鳞状细胞癌；KICH：肾嫌色细胞癌；KIRC：肾透明细胞癌；KIRP：肾乳头状细胞癌；AML：急性髓系白血病；LGG：低级别脑胶质瘤；LIHC：肝癌；LUAD：肺腺癌；LUSC：肺鳞癌；MESO：间皮瘤；OV：卵巢浆液性囊腺癌；PAAD：胰腺癌；PCPG：嗜铬细胞瘤和副神经节瘤；PRAD：前列腺癌；READ：直肠腺癌；SARC：肉瘤；SKCM：皮肤黑色素瘤；STAD：胃癌；TGCT：睾丸癌；THCA：甲状腺癌；THYM：胸腺癌；UCEC：子宫内膜癌；UCS：子宫肉瘤；UVM：葡萄膜黑色素瘤

2. hnRNP U在AML患者中表达：为研究hnRNP U在AML患者中的表达情况，收集苏州大学附属第一医院血液科及皖南医学院第二附属医院血液科2021年11月1日至2022年2月11日收治的26例AML患者血液样本，27名健康对照者血液血液样本，分离出外周血单个核细胞（PBMC）后提取RNA，检测hnRNP U的表达水平，结果如图2所示，hnRNP U在AML患者中的mRNA表达水平显著高于健康对照者（0.0315±0.0042对0.0195±0.0006，*P*<0.01）。另取其中3例AML患者及1名健康对照者的PBMC收集蛋白，通过Western blot法检测hnRNP U的蛋白表达，结果显示hnRNP U在AML患者中的蛋白表达水平高于健康对照者（图2）。

**图2:**
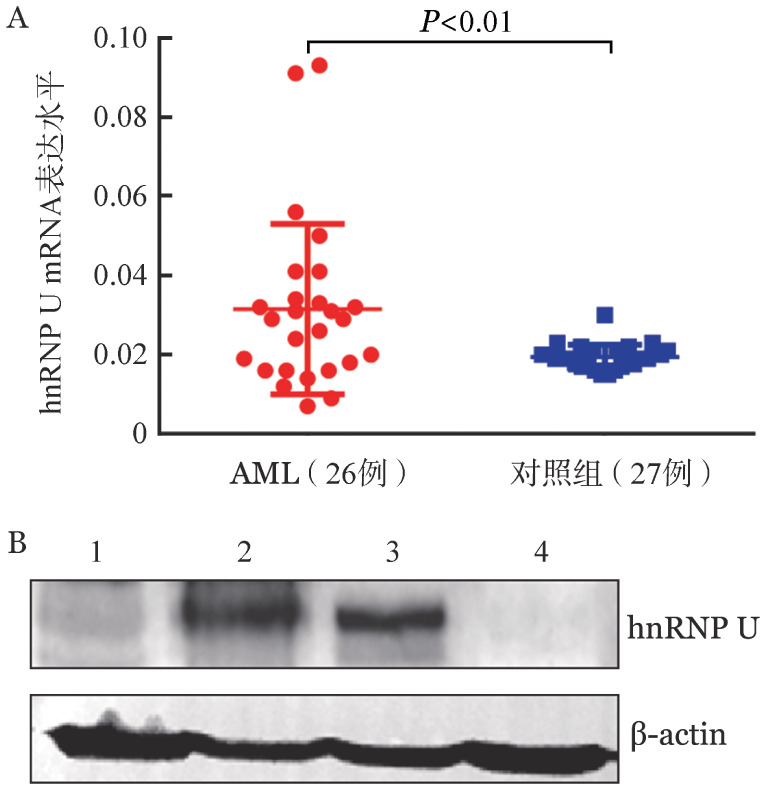
急性髓系白血病（AML）患者与健康对照者外周血单个核细胞中hnRNP U mRNA表达（A）和蛋白表达（B） 1～3：AML患者；4：健康对照者

二、hnRNP U与AML临床特征的相关性

通过cBioPortal数据库（http://www.cbioportal.org）下载Beat AML数据集临床信息及转录组数据，并进行数据清洗，剔除非AML、无RNA测序数据病例以及无药物敏感性数据后得到158例AML患者数据，按照hnRNP U表达水平分为高表达组（89例）和低表达组（69例），通过比较hnRNP U高表达组和低表达组的临床特征，发现hnRNP U高表达组较hnRNP U低表达组发病年龄更早，且合并FLT3突变比例更高，差异均有统计学意义（*P*值均<0.05）（表3）。

**表3:** hnRNP U表达与急性髓系白血病（AML）患者临床特征相关性分析

临床特征	hnRNP U高表达组（89例）	hnRNP U低表达组（69例）	统计量	*P*值
性别（例，男/女）	41/48	42/29	3.415	0.065
年龄[岁，*M*（范围）]	56（2~87）	65（8~85）	−2.681	0.007
WBC[×10^9^/L，*M*（范围）]	45（2~427）	29（2~250）	−1.595	0.111
HGB[g/L，*M*（范围）]	10（5~15）	8.5（5~15）	−0.236	0.813
PLT[×10^9^/L，*M*（范围）]	39（6~228）	46（4~916）	−1.017	0.309
ELN2017危险度分层[例（%）]			1.566	0.457
低危	34（38.2）	21（30.4）		
中危	28（31.5）	21（30.4）		
高危	27（30.3）	27（39.2）		
诱导化疗反应[例（%）]^a^			2.211	0.137
CR	57（77.0）	29（64.4）		
NR	17（22.0）	16（35.6）		
FLT3突变[例（%）]			4.069	0.044
阳性	28（31.5）	12（17.4）		
阴性	61（68.5）	57（82.6）		
NPM1突变[例（%）]			1.389	0.239
阳性	31（34.8）	18（26.1）		
阴性	58（65.2）	51（73.9）		

注：CR：完全缓解；NR：未缓解。^a^hnRNP U高、低表达组分别有74和45例评估诱导化疗反应

三、hnRNP U对AML细胞系生物学行为的影响

1. hnRNP U在AML细胞系中表达情况：检测AML细胞系U937、OCI-AML3、THP-1、MV4-11、MOLM-13、Kasumi-1中hnRNP U蛋白表达水平，其中Kasumi-1、MOLM-13细胞的hnRNP U蛋白水平偏高，U937和OCI-AML3细胞的hnRNP U蛋白表达水平偏低（图3），因此选取Kasumi-1和MOLM-13细胞作为研究hnRNP U在AML中致病机制的工具细胞。

**图3:**
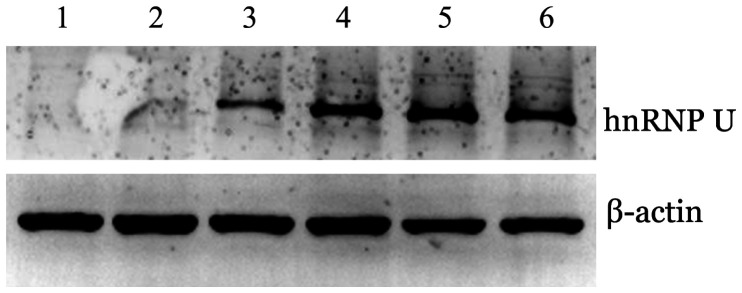
Western blot法检测急性髓系白血病细胞系中hnRNP U蛋白表达水平 1：U937细胞；2：OCI-AML3细胞；3：THP-1细胞；4：MV4-11细胞；5：MOLM-13细胞；6：Kasumi-1细胞

2. 成功构建敲低hnRNP U的细胞系：感染72 h后GFP蛋白可在转染成功的细胞中表达，用流式细胞仪检测GFP阳性细胞占全部细胞的比例即为感染效率。感染后72 h，从转录水平出发，与shscramble组相比，Kasumi-1细胞的shhnRNP U#1、shhnRNP U#2组敲低效率分别为89.95％和91.14％，MOLM-13细胞的shhnRNP U#1、shhnRNP U#2组敲低效率分别为86.25％和68.91％；从蛋白水平出发，与shscramble组相比，Kasumi-1细胞和MOLM-13细胞的hnRNP U蛋白水平均显著下调（图4）。

**图4:**
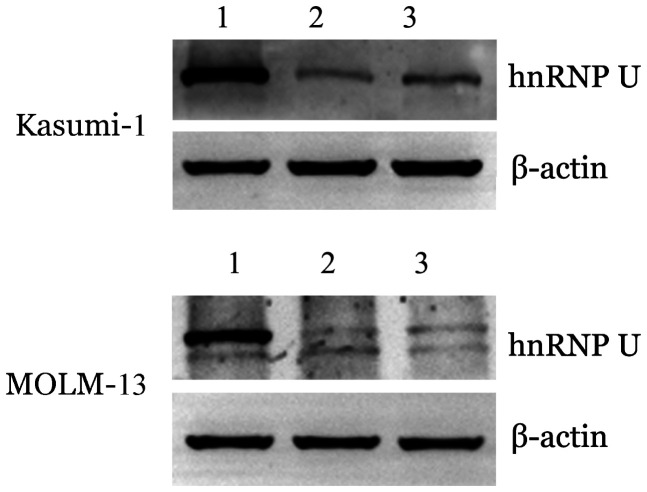
Western blot法检测在Kasumi-1和MOLM-13细胞中敲低hnRNP U后hnRNP U表达水平的变化 1：对照组；2、3：分别为shhnRNP U#1、shhnRNP U#2组

3. 敲低hnRNP U后对细胞增殖能力影响：细胞增殖反应曲线见图5。敲低hnRNP U后Kasumi-1和MOLM-13细胞的增殖能力显著减弱。

**图5:**
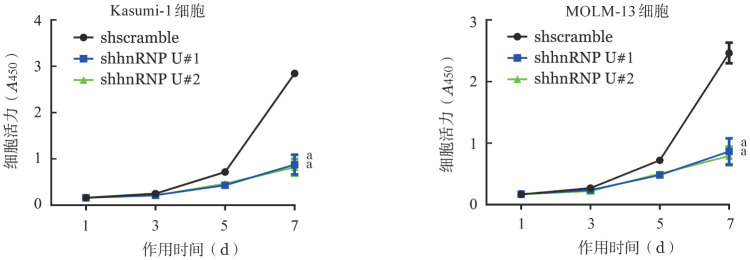
CCK-8检测在Kasumi-1和MOLM-13细胞中敲低hnRNP U对细胞增殖能力的影响（实验重复3次） 与对照组（shscramble）比较，^a^*P*<0.001

4. 敲低hnRNP U对细胞凋亡影响：Annexin Ⅴ-APC阳性细胞和7-AAD阳性细胞占总细胞数的比例即为细胞凋亡率。如图6所示，敲低hnRNP U后Kasumi-1和MOLM-13细胞的凋亡率显著升高。

**图6:**
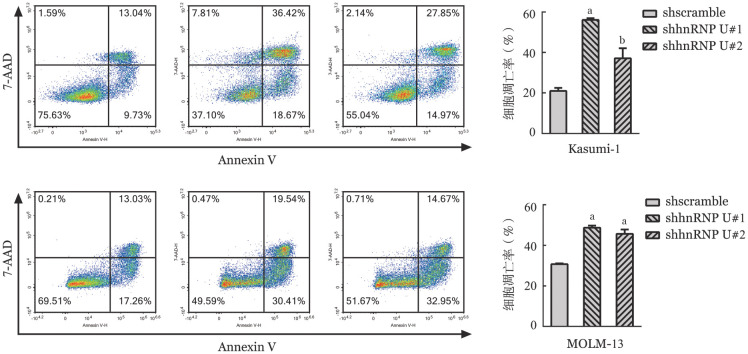
Annexin Ⅴ-APC/7-AAD双染色流式细胞术分析Kasumi-1和MOLM-13细胞敲低hnRNP U后的细胞凋亡率（实验重复3次） 与对照组（shscramble）比较，^a^*P*<0.001，^b^*P*<0.01

5. 敲低hnRNP U对细胞周期影响：转染后第5天细胞周期各阶段细胞比例见图7。与shscramble组相比，Kasumi-1和MOLM-13细胞敲低hnRNP U后G_2_/M期的细胞比例增高，表明敲低hnRNP U可使AML细胞阻滞于G_2_/M期。

**图7:**
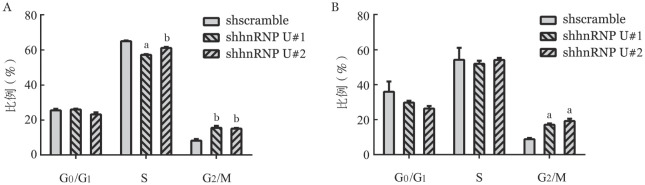
流式细胞术检测在Kasumi-1（A）和MOLM-13细胞中（B）敲低hnRNP U对细胞周期的影响（实验重复3次） 与对照组（shscramble）比较，^a^*P*<0.01，^b^*P*<0.05

6. 敲低hnRNP U对细胞集落形成能力影响：与shscramble组相比，敲低hnRNP U后Kasumi-1和MOLM-13细胞的集落形成能力明显减弱（图8）。

**图8:**
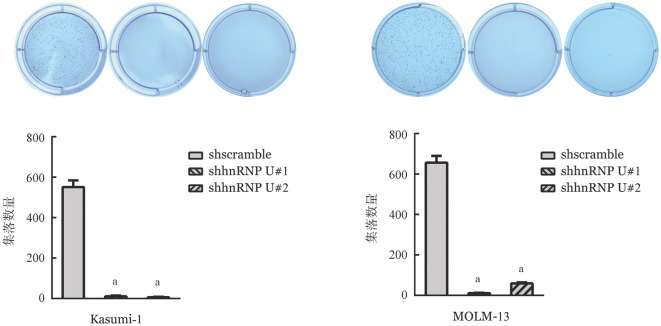
软琼脂集落形成实验检测在Kasumi-1和MOLM-13细胞中敲低hnRNP U后细胞集落形成能力变化（实验重复3次） 与对照组（shscramble）比较，^a^*P*<0.001

7. 敲低hnRNP U对细胞DNA损伤应答（DDR）通路影响：利用Western blot法检测发现，Kasumi-1和MOLM-13细胞敲低hnRNP U后PARP剪切体形式增加，同时p-H2A.X蛋白表达量升高（图9）。

**图9:**
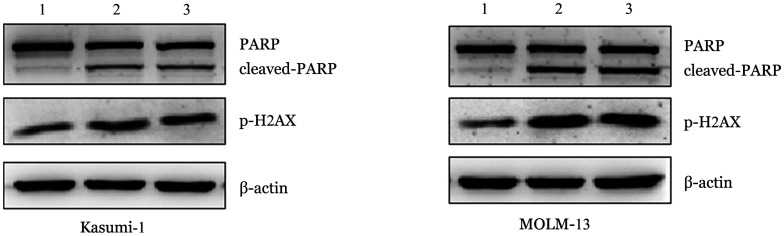
Western blot法检测在Kasumi-1和MOLM-13细胞中敲低hnRNP U后DDR通路蛋白表达变化 1：对照组；2：shhnRNP U#1组；3：shhnRNP U#2组

## 讨论

AML是成人中常见的血液系统恶性疾病，好发于中老年，具有高度分子学及临床异质性[5]–[7]。目前人类对AML的发病机制尚不完全清楚，现认为是由多种因素相互作用而导致的，因而积极探索AML发生发展的分子机制，寻找新的诊断标志物和治疗靶点具有重要的临床意义。

hnRNP U是近几年备受关注的肿瘤相关基因，定位于人类染色体1q44位点，含有14个外显子，最早在HeLa细胞中发现，该基因编码的蛋白可以与支架附着区（Scaffold attachment region，SAR）DNA分子结合，因此也被称为支架附着因子A（Scaffold attachment factor A, SAF-A）[8]。hnRNP U属于核不均一核糖核蛋白家族的重要一员，是染色体结构重塑及维持染色质3D结构所必须的一个蛋白分子[9]–[10]，还可以与端粒特异性结合，维持端粒的“酶活性”[11]，hnRNP U能够与Xist蛋白稳定结合，协助Xist蛋白附着在X染色体上，致使X染色体失活[12]–[13]。因此，hnRNP U对维持细胞内生命活动的进行有着不可替代的作用。在病毒RNA侵入宿主后，hnRNP U识别病毒RNA，调节染色质重构以及基因增强子活性来提高细胞抗感染基因的表达[14]，同时hnRNP U在调节RNA稳定性[15]、参与前体mRNA选择性剪切[16]、细胞有丝分裂过程[17]、RNA加工修饰运输、与RNA聚合酶Ⅱ结合从而调控转录翻译[18]生物学过程中也发挥了重要的作用。hnRNP U的异常表达与多种癌症的发生发展及患者的不良预后相关，据文献报道，在肝癌细胞中，DIS3L2通过CSD结构域招募HNRNP U，介导其与前Rac1产生相互作用，从而产生Rac1b剪接异构体，促进肝癌进展[19]；在胃癌中hnRNP U通过与HPSE的RNA结合促进其与组蛋白乙酰化酶p300在增强子上的相互作用，导致p300介导的EGR1反式激活，促进靶基因表达从而促进胃癌的恶性病程[20]；在神经母细胞瘤中，hnRNP U促进细胞核因子HNF4A-AS1与CCCTC结合因子CTCF结合，从而促进神经母细胞瘤细胞的生长和侵袭[21]。迄今为止，在血液系统肿瘤，尤其在AML中，hnRNP U调控的生物学过程报道较少。本课题组通过探讨hnRNP U在AML中的临床意义发现hnRNP U高表达组较hnRNP U低表达组发病年龄更早（*P*＝0.007），且更容易合并FLT3突变（*P*＝0.044）。其中，FLT3突变是AML高危的指标，这提示我们hnRNP U高表达与AML发生发展相关联，但两者相关性仍需要进一步的机制研究。

本研究通过体外功能实验，发现敲低hnRNP U的表达会抑制AML细胞增殖，促进其凋亡，阻止细胞周期的正常进行，提示hnRNP U高表达可通过促进AML细胞增殖从而促进疾病侵袭性增加、耐药等恶性病程。Western blot检测发现敲低hnRNP U后可引起PARP剪切体形式增加，p-H2A.X蛋白表达量升高，DDR[22]是指因诱导和检测DNA损伤而导致的细胞内和细胞间不同信号转导事件和酶的激活，Cleaved-PARP和p-H2A.X蛋白是DDR通路上的关键蛋白[23]，其表达量增加可反映复制叉压力增加和DNA损伤程度增加，这提示我们对于hnRNP U高表达的AML可通过给予拓扑异构酶抑制剂等化疗药物引起hnRNP U表达下调，进而引起DNA损伤程度增加，诱导AML细胞凋亡的发生。

综上所述，hnRNP U在AML中高表达且与AML的发生发展相关，这对于AML的早期诊断、治疗以及提高患者生存质量具有重要意义。但对于hnRNP U在AML中诱导DDR反应的具体机制，仍有待进一步研究。相信随着对AML发生机制研究的不断深入，AML的靶向治疗将会迎来新的突破。
